# The life-cycles of skin replacement technologies

**DOI:** 10.1371/journal.pone.0229455

**Published:** 2020-03-04

**Authors:** Mihail Climov, Adriana C. Panayi, Gregory Borah, Dennis P. Orgill

**Affiliations:** 1 Division of Plastic Surgery, Ruby Memorial Hospital, West Virginia University, Morgantown, WV, United States of America; 2 Tissue Engineering and Wound Healing Laboratory, Division of Plastic Surgery, Brigham and Women’s Hospital, Harvard Medical School, Boston, MA, United States of America; 3 Division of Plastic Surgery, University of New Mexico School of Medicine, Albuquerque, NM, United States of America; Universite de Technologie de Compiegne, FRANCE

## Abstract

**Introduction:**

Skin Replacement Technologies (SRTs) emerged as skin alternatives for burns, large excisions or trauma. The original publications represent the available knowledge on a subject and can be modeled as a logistic S-curve which depicts the technology’s evolution life-cycle. The Technology Innovation Maturation Evaluation (TIME) model was previously introduced to study the life-cycles of biotechnologies.

**Methods:**

PubMed database was searched 1900–2015 to review relevant publications. All skin replacement or regeneration products on the US market were included. The TIME model was applied to assess evolutionary patterns for each technology.

**Results and discussion:**

Three SRT clusters were identified: processed biologics technologies (PBT), extracellular matrix technologies (EMT), and cell-based technologies (CBT). Publications on EMTs and CBTs start decades after PBTs, however, are greater in number and follow an ascending trend. PBTs reached a plateau, suggesting near-senescence. The CBT curve was non-logarithmic and the TIME model could not be applied. The technology initiation point (T_i_) for PBTs was 1939 and the establishment point (T_e_) 1992. For EMT, T_i_ was 1966 and T_e_ 2010. Sixty-one products were identified (49 EMTs, 7 CBTs, 5 PBTs). PBTs appeared 11 years after T_e_ and EMTs four years prior T_e_. Thirty-seven products in the EMT category, and one in the PBT category, were developed before T_e_. The most common FDA regulatory mechanism for SRT was found to be 510(k) followed by HCT/P 361.

**Conclusion:**

Innovation is an indicator of the evolution of technology. The number of publications can be used as a metric of this evolution and the fact that the SRT field falls under such pattern demonstrates that SRT is an innovation-based industry. EMT is the most efficient cluster. Few products from SRT registered a commercial success, and from those that did, those technologies were generally found to be part of the most productive cluster, 1^st^ in concept, conceptually simple, easily regulated and produced, cost and clinically efficient, reimbursable, able to solve a specific problem efficiently, had a platform technology design that allowed for further innovation and adaptation for other uses and, as found by application of the TIME model, appear prior to technology establishment.

## Introduction

Technological advancement and proper management are imperative for the success of new products, and awareness of technology life cycles (TLC), defined as a product’s commercial gain and financial return during its life span from research and development to market maturity to decline, is becoming increasingly important for medical professionals[1]. Knowledge can aid prediction and projection of product potential as well as the successful establishment of innovation avenues.

Prior research showed that TLCs follow a sigmoid or S-curve [2]. Following the development of a technology, there is an initial period of slow growth and knowledge expansion during which time the prototypes are tested. The innovation then undergoes improvements characterized by linear growth. Eventually, the innovation attains maturity, and at this time products are expected to debut on the market. After this stage, there is minimal innovation and the product either enters a stagnation or a decline phase. Factors that influence the length of each phase of the cycle include the inherent characteristics of the product, its management, as well as external market conditions [3]. The standard assumption is that the natural course of all technologies is to reach a plateau followed by a period of senescence and obsoletion or a technological jump with the start of a new cycle [4].

Publications on medical technologies have been shown to exponentially accumulate and can be depicted on a sigmoid curve. Statistical analysis of a number of PubMed entries using the Technology Innovation Maturation Evaluation (TIME) model has been used to simulate the technological growth and maturation of biotech products [5]. Successful products dependent on the degree of technological maturity and studying the correlation between inflection points along with the elements of technological productivity provides useful insights for product management as well as the projection of future developments [6]. Application of the TIME model [6] to the fields of gene therapy, Alzheimer’s disease and cancer therapeutics [7–9] highlighted the main milestones in publication accumulation, specifically the point of initiation, T_i_—occurrence of seminal events that enable exponential growth of the literature—and the establishment point, T_e_—when exponential growth starts to slow down. Prior research using the TIME model has shown that a period of 14 years for cancer therapeutics, [9] 22 years for Alzheimer’s disease [7], 31 years for cardiovascular[10], and 25 years in general for translational science [6] must pass after T_e_ for products to be developed. Although new technologies bring great promise, they seldom meet the market standards resulting in failure [6–9]. Such exits are enforced by currently established technologies [4], and only technologies that manage to achieve some level of maturity remain successful [6].

Given that skin is the largest organ of the body and a 30% loss has the potential to be lethal, there has been extensive innovation in the development of Skin Replacement Technologies (SRTs). These technologies are life-saving, particularly when autologous tissue is unavailable. Mortality due to extensive burns has dramatically decreased since the 1950s, partly due to advancements in intensive care but also SRT development [11–14]. Despite the fact that skin was the first human tissue to be “engineered,” there is little understanding of SRT product development, TLCs and why this technology represents a slowly evolving industry.

SRTs can be broadly subcategorized into Processed Biologics Technologies (PBTs), Extracellular Matrix Technologies (EMTs) and Cell-based Technologies (CBTs). PBTs represent the oldest type of SRT and include technologies for harvesting, sterilizing, processing and preserving skin grafts to enhance infection control and off-the-shelf lifespan. Although allo- and xenografts have been used for a long time, the first publications on the technology, detailing issues of graft rejection, came only at the end of the 19^th^ and beginning of the 20^th^ century.[15,16] Later publications described early attempts to decrease rejection[17–19] EMTs are technologies that provide a scaffold that can be incorporated into the body by engraftment and angiogenesis helping to replace or regenerate the dermis.[20–23] These technologies consist of natural biomaterials, decellularized biologic tissues, or semi-synthetic materials. The first in concept EMT is Integra® (Integra LifeSciences Corporation, Plainsboro, NJ), a collagen-glycosaminoglycan scaffold used as a dermal replacement (Table 1).

**Table 1:** Technological clusters with first in concept products.

Technology	Description	Representative	1^st^ in concept product	Time difference since 1^st^ historical publication and FDA clearance (years)	1^st^ historical publications	FDA clearance of 1^st^ in concept NTE
**Processed biologics technologies (PBT)**	Technologies that use minimal processing (freezing, irradiating, lyophilizing) allogeneic or xenogeneic skin for integument replacement or regeneration	Skin Allograft	Irradiated human allograft (Gammagraft)	136	1869 Reverdin[16,36,50,51]	2005 does not required FDA clearance (PHS 361)
		Skin Xenograft	Frozen xenograft (Mediskin)	>3400	1500 BC. [52]	1983 cleared by FDA, currently not present on the market
**Extracellular matrix technologies (ECMT)**	Technology that uses scaffolds mimicking the extracellular matrix, for skin or skin components substitution/regeneration obtained by decellularization or biosynthesis from natural or synthetic materials	Biosynthetic scaffolds	Collagen-Condroitin scaffold (Integra)	15	1981[23,53]	1996 (PMA)
		Decellularized tissues (mostly cadaveric, porcine or bovine dermis, also pericard, small intestine submucosa, urinary bladder etc.)	Decellularized human dermis (AlloDerm)	-1	1995[54]	1994 (510k)
**Cell-based technologies (CBT)**	Technology that uses living cells of different origins for skin substitution or regeneration (may also use scaffolds or hydrogels)	Cultured Epithelial Autografts	Cultured Epithelial Autograft (Epicel)	17	1981[25]	1998 (PMA)
		Allogeneic bilayered skin constructs	Allogeneic dermo-epidermal skin construct (Apligraf)	17	1981[27]	1998 (PMA)
		Autologous bilayered skin constructs	Autologous Dermo-Epidermal skin construct (Cultured Skin Substitute, NovaDerm, Permaderm)	Not cleared yet	1995[32]	2007 (IND), currently not cleared by FDA

**Fig 1:**
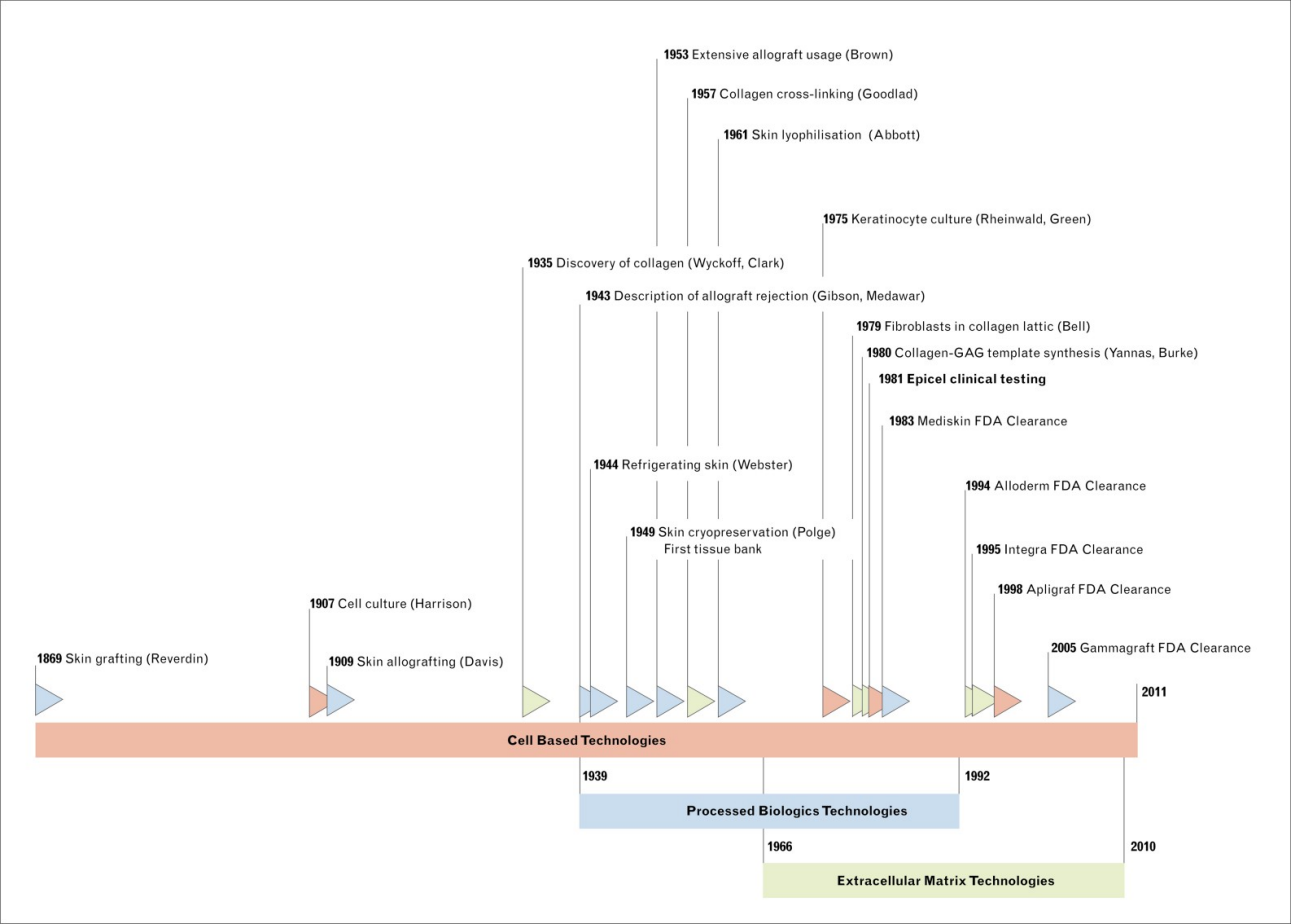
SRTs in a historical context highlighting significant milestones.

Matrix decellularization is another approach to obtain a scaffold that naturally mimics the dermal extracellular matrix (ECM) but lacks cells consequently limiting rejection as the most immunogenic components are removed. The first product to be developed was AlloDerm® (LifeCell Corporation, Branchburg, NJ). Similar processes were applied to other tissues, including small intestinal submucosa and pericardium, from various sources such as cadavers, bovine, porcine, and neonatal animals. This has resulted in various products with different applications (Table 1, Fig 1).

CBTs arose with the introduction of an improved method of culturing keratinocytes allowing the production of large epithelium sheets for grafting.[24] Cultured epidermal autografts were first used in the treatment of major burns in 1981.[25] The technology led to the commercially available product Epicel^™^ (Vericel Corporation, Cambridge, MA). Other approaches were tested, most notably the “Living Skin Equivalent”- a bilayer of fibroblasts in a collagen lattice and an epidermal-like layer constructed from keratinocytes. Although promising, this failed to engraft.[26–29] A variation of this product was commercialized as the allogeneic bilayered equivalent-Apligraf® (Organogenesis Inc, Canton, MA) that works mostly as a biologic dressing converting a chronic to an acute wound.[30] EMTs were subsequently combined with cells [31,32] leading to several technologies, including NovaDerm^™^, StrataGraft®, Dermagraft®, and OrCel®, all in different stages of FDA clearance and marketing (Fig 1, Table 1).

In this study, we applied the TIME model to SRTs to establish the pattern of evolution in this sector. Our goal is to elucidate whether the field follows the previously described model of growth, quantify the capacity for innovation, and attempt to correlate this with the economic and clinical efficiency of products. Our hypothesis is that one of the characteristics that predict the success of an SRT is the capacity to allow for further innovation.

## Results

### SRT classification

Given the large heterogenicity of products, we classified SRTs into three clusters according to their method of action, degree of processing and source: Processed Biologics Technologies (PBT), Extracellular Matrix Technologies (EMT), and Cell-Based Technologies (CBT). First-in-concept products are highlighted for each cluster (Table 1). All three clusters were found to be disruptive to the prior standard of care, that is moist-wound therapy.

### Application of theories of innovation to SRTs

**Fig 2:**
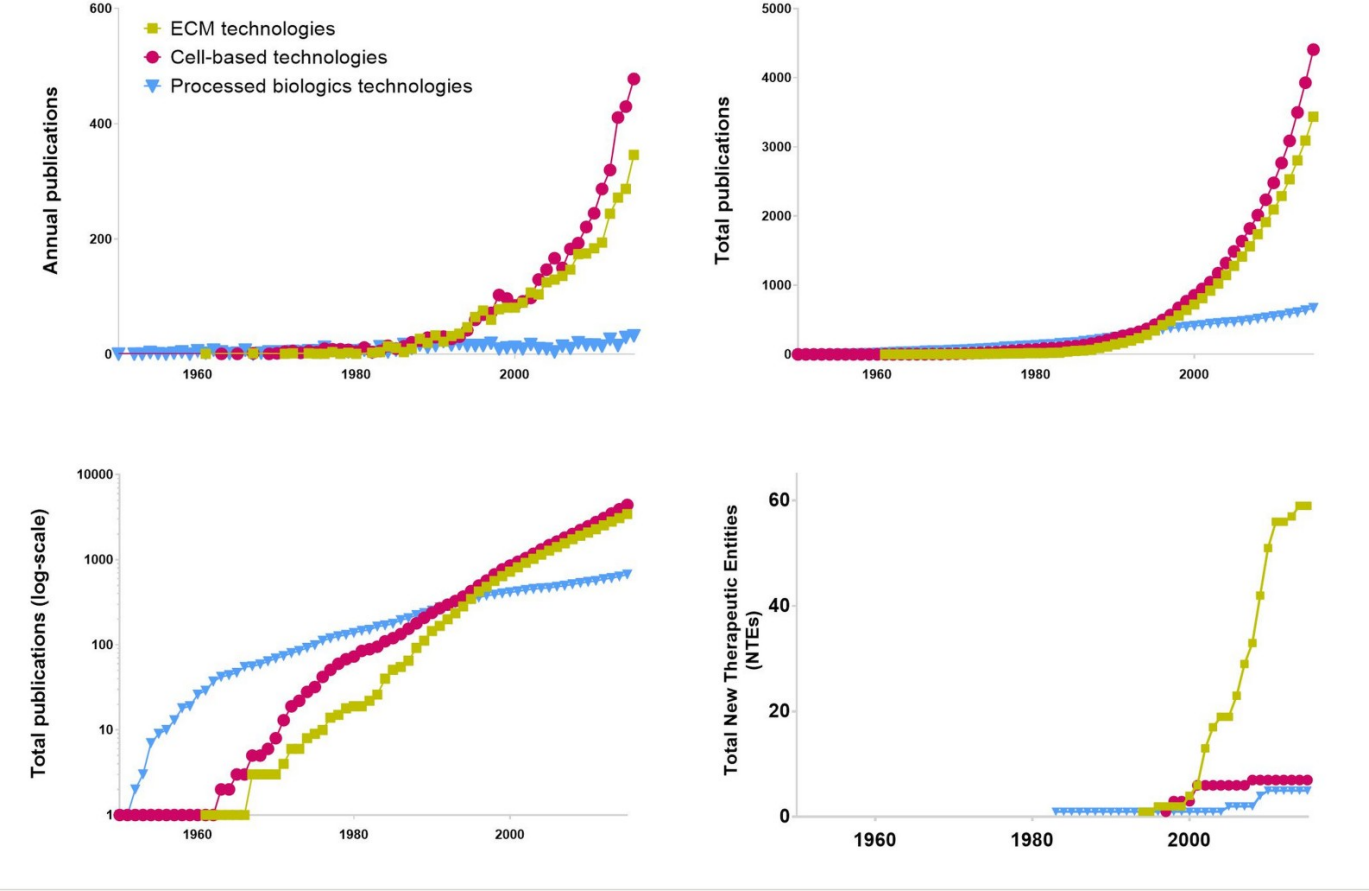
Quantification of publications and new therapeutic entities for SRTs.

**Fig 3:**
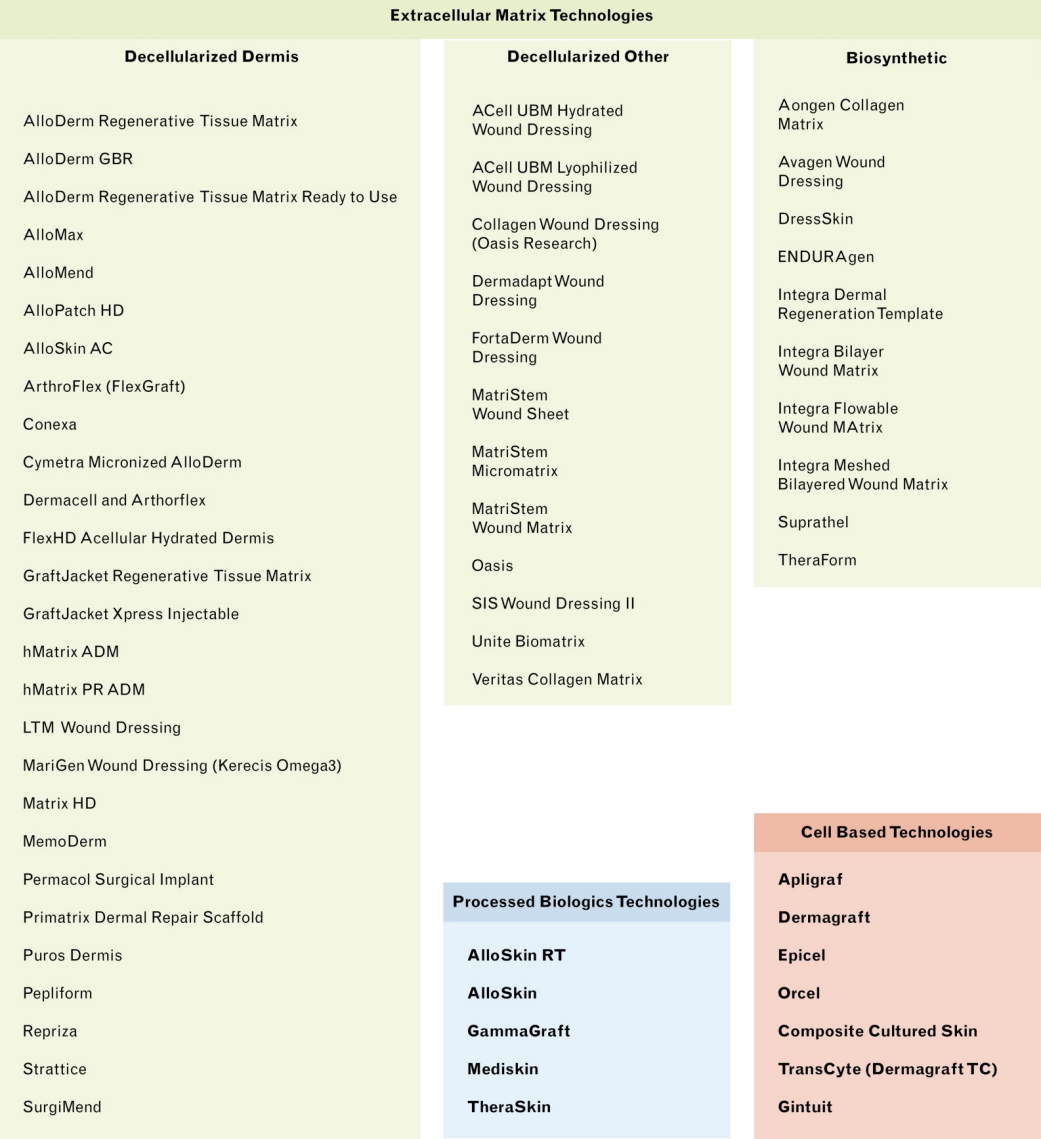
SRT products analyzed and included in this research.

**Fig 4:**
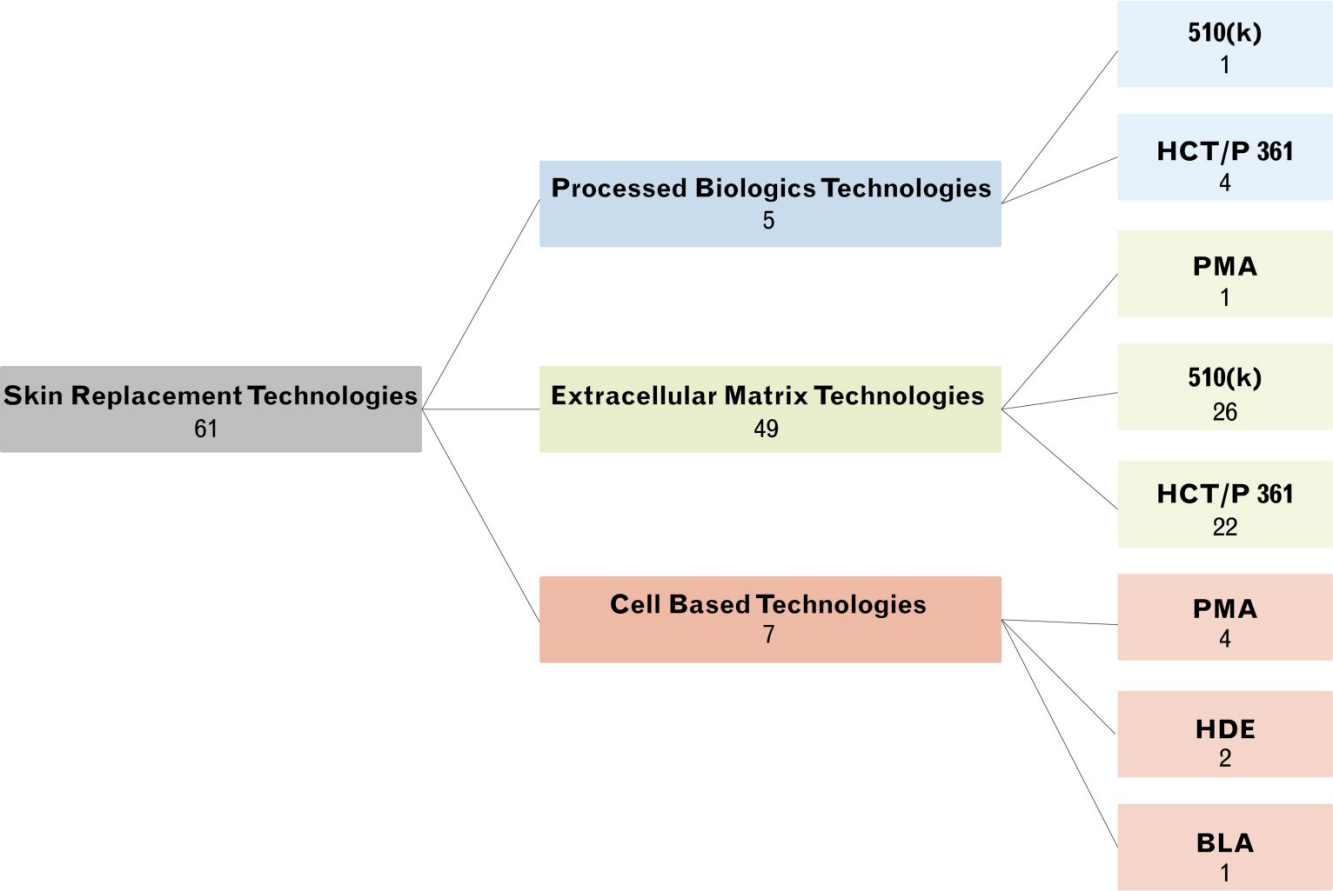
SRT products classified according to regulatory mechanism.

Analysis of publications emphasized that number increases with time in all clusters. Publications on EMTs and CBTs start several decades after the first PBT publication (Fig 2 and S2 Fig). Despite this, EMTs and CBTs have a larger number of original publications and continue to follow an ascending trend. Overall 61 products satisfied the criteria to be considered an SRT, with the most productive cluster being EMTs (49), followed by CBTs (7) and finally PBTs (5) (Figs 3 and 4).

**Fig 5:**
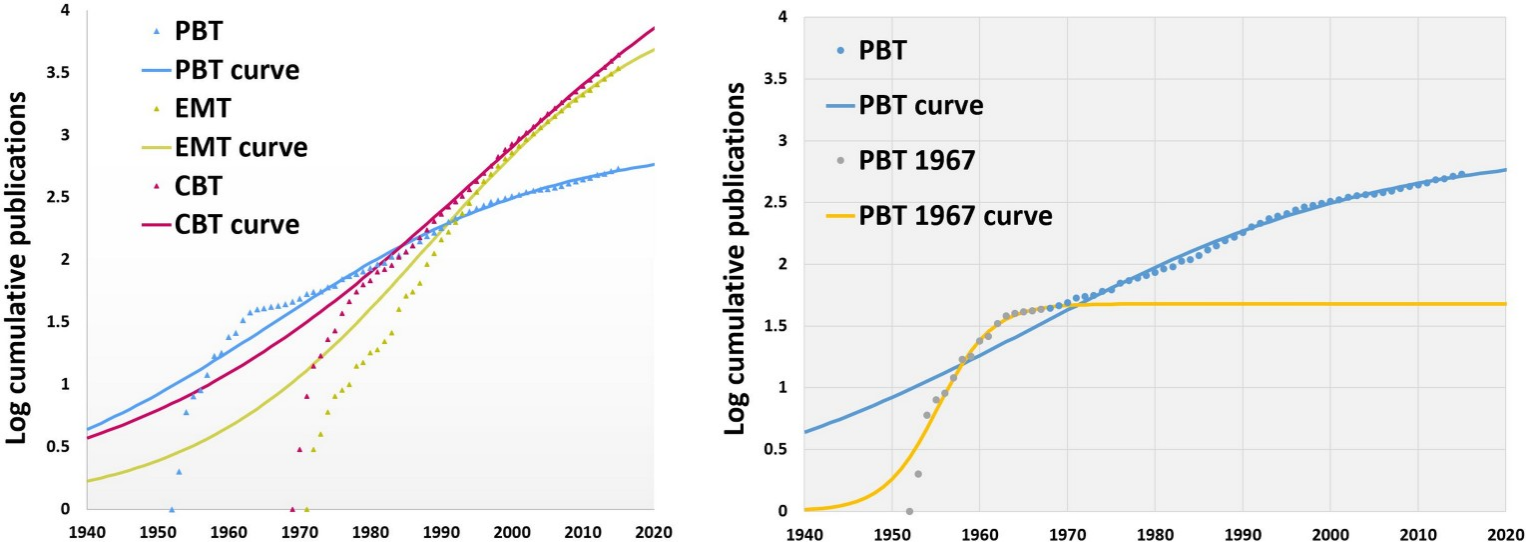
Technology life-cycle curve of SRTs. A (left). Initiation and establishment points were calculated as previously published.[7] B (Right). PBT curve presents with several inflection points, one in 1968 and second in 2005. The 1^st^ inflection point corresponds with a small technological jump that is likely associated with the series of inventions and studies on allograft cryopreservation, use for burns, as well as the increasing use of tissue banks. After 1967, there is a spike in publications correlating to multiple studies attempting to alter skin immunogenicity as well as clinical studies investigating the use of allografts in burns. The jump seen in 2005 may be associated with the first face transplant that put in the spotlight the problem os skin rejection and attempts to overcome.

**Fig 6:**
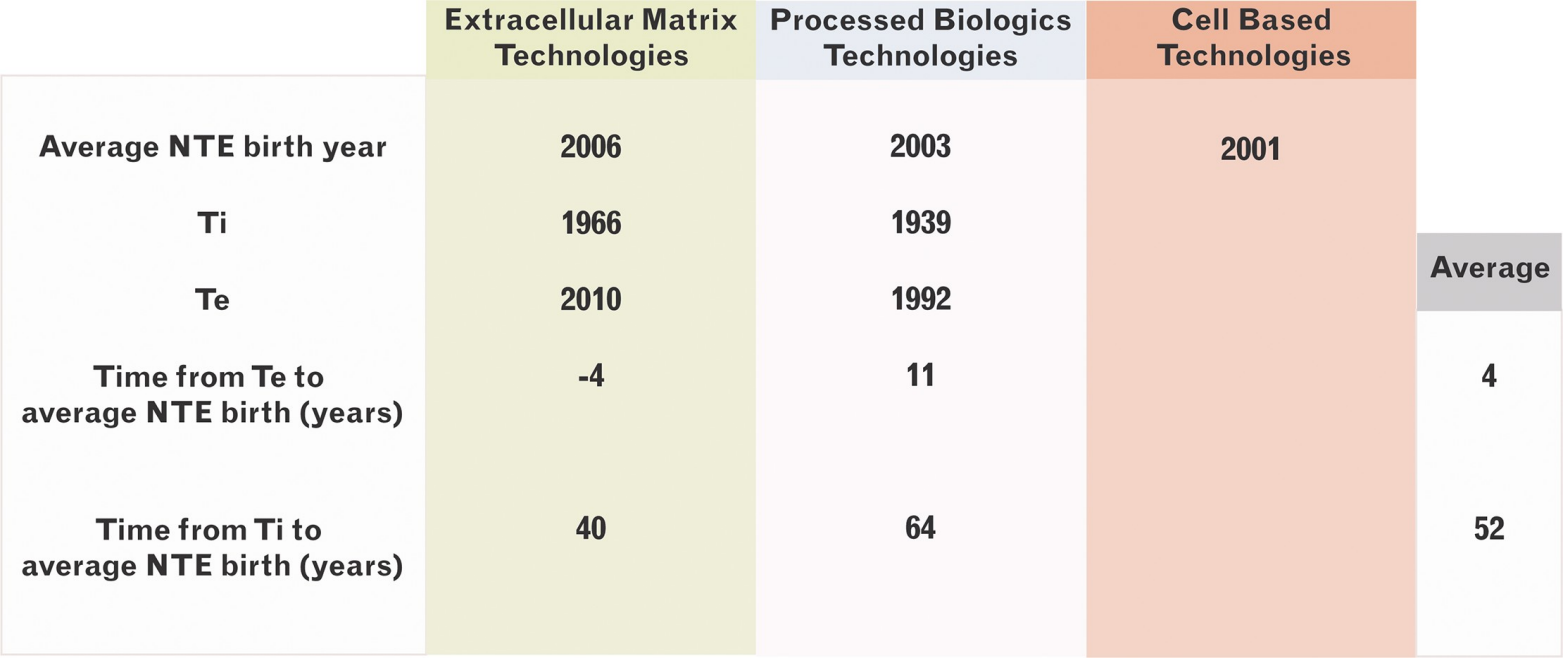
TIME model analytics. Emphasized technology initiation and establishment timepoints.

The T_i_, where linear growth starts, for PBTs was 1939 which corresponds to increased use of skin allografts during the war, that later continued with the work of Billingham and Medawar on skin transplantation,[33,34] as well as the early clinical studies on allografts and xenografts.[19,35,36] Eventually exponential growth slowed down, reaching the T_e_ in 1992 after which publications significantly decreased. There are two inflection points, 1967 and 2005. A boost in innovation occurred in 1967 causing the curve to display a technological jump (Figs 1, 5 and 6). Associated with this period are inventions and studies on allograft cryopreservation, use for burns, as well as the increasing use of tissue banks. After 1967, there is a spike in publications correlating to multiple studies attempting to alter skin immunogenicity as well as clinical studies investigating the use of allografts in burns. The jump seen in 2005 may be associated with the first face transplant that drew attention to the subject of skin rejection. In the same year, Gammagraft®, an irradiated skin allograft product, received FDA clearance enabling longer storage and applications that do not require freezing.

PBTs are not productive from a New Therapeutic Entity (NTE) perspective, as hospital-based tissue banks, and cadaveric labs decreased the need for commercially available products. Despite the widespread use of PBTs from the start of the century, the first commercially available product, Mediskin, a porcine frozen skin xenograft, was only approved in 1983 and is no longer available. In this category, all products appeared after the T_e_ (1992) with the exception of Mediskin. The technology evolution curve plateaus after 1992 suggesting that the cluster has reached its limit and is approaching senescence (Figs 5 and 6). It should be noted that although allografts and xenografts were in use much earlier than the calculated T_e_ they were not adopted by industry, possibly due to lack of technological infrastructure.

The T_i_ for EMTs was in 1966, which corresponds with extensive work on the function of collagen in the dermis as well as on altering its composition. Studies included decellularization experiments and development of freeze-drying following by a period of exponential growth of publications was seen. T_e_ was reached in 2010 a time with an advanced understanding of collagen and other biomaterials as well as their interaction with wounds, their porosity, structure and other properties. Thirty-seven NTEs were developed before T_e_, with first in concept approved by FDA being AlloDerm^™^ in 1994 and Integra® in 1996. Integra® was already in experimental use as early as 1981, but similarly to Epicel^™^, it received FDA approval much later.

The T_i_ for CBTs was difficult to establish as the curve is still growing and does not appear to follow the same pattern. This cluster has not yet reached its T_e_, which is encouraging for future research especially in the development of an allogeneic, “off-the-shelf,” model. Although few NTEs have been approved, all appear before T_e_. First in concept is Epicel^™^ that has been in use since 1981. This is the first cellular product to be used in humans and the only currently approved permanent solution for large skin defects. Eight NTEs were included in the CBT cluster, and only Epicel^™^, Apligraf®, and Dermagraft® are on the market. OrCel®, Transcyte®, and Gintuit^™^ failed despite being hailed as promising products.[37–40] NovaDerm^™^ and StrataGraft® failed to receive FDA approval and did not reach commercialization so far, despite StrataGraft® completing Phase III of clinical trials[41] and NovaDerm^™^ showing successful results in a clinical trials.[42] Apligraf® was initially developed for use in burns or reconstruction, but showed unexpected results in trials and is now used as a temporary, bioactive wound dressing for chronic wounds.

### Regulation in SRT evolution

In our analysis, we identified two major pathways of FDA clearance for SRT products. PHS 361 that does not require FDA pre-approval but follows rigorous manufacturing guidelines, good tissue practice and procedures to prevent infectious diseases and PHS 351 that requires FDA preapproval and has five licensing options: 1) Premarket application (PMA): requires proof of safety and effectiveness of class III devices, 2) Humanitarian Device Exemption (HDE): does not require proof of efficacy[43, 3] 510(k): premarket submission that shows that the device is as safe and effective as a legally marketed product, 4) Biologics License Application (BLA): clearance mechanism for biologicals. Only Gintuit^™^ was cleared through this mechanism, 5) NDA (new drug application): no NTEs are regulated through this mechanism.

SRTs were classified according to the regulatory mechanism (Fig 4). EMTs had the easiest regulation: PHS 361 (n = 22) and 510(k) (n = 26). Although the straightforward production—in terms of processing and materials—of EMTs and PBTs is more attractive to industry, the development, clinical trials, and commercialization costs are lower than PMA cleared products. The extent of processing, as well as the materials, influence what regulation may be required. PMA products have narrow applications that are highly enforced and regulated, unlike 510(k) products. Two CBTs (NovaDerm^™^, StrataGraft®) have an orphan status designation, which offers multiple incentives including tax credits for clinical testing [44].

## Discussion

Success, in broad strokes, means achieving a goal, and in business, signifies monetary gain.[45–47] In biotech, success requires clinical and business effectiveness. We support that SRTs are clinically successful when they offer a significant advantage over pre-existing products, and successful in business when they continuously generate profits as well as provide the potential for technological innovation.

In our analysis, we found that the prerequisites for success include maturity, the potential for innovation, easy regulation. Efficiency, that is the ability to offer better treatment, is related to reimbursement. EMTs were the most efficient cluster and had 37 NTEs approved prior to T_e_. This questions whether publication numbers accurately mark success in this industry. Skin allografts and xenografts had been in use since ancient times, however, only recently became commercialized. One of the earliest developments, Epicel^TM^, does not have the highest financial success, possibly due to inadequate market size as it addresses only a small fraction of the population. Epicel^TM^ has a prohibitive construction cost as the autologous approach business model is complex and requires preliminary skin biopsy and culture.

The two most successful SRTs, AlloDerm^™^ and Integra®, have allowed further improvement and experimentation. This emphasizes the importance of successful marketing and management, active research and good development team. Although both started as SRTs, AlloDerm^™^ was found to be more useful clinically as a mesh for breast or hernia surgery. Consequently, finding other applications for the technology as well as designing a technology that allows use in other areas in our assessment is a predictor of success. Innovation should not be limited to the intended purpose. This was seen with Integra® when its intended use was extended from burns to chronic wounds and nerve regeneration. When quantifying publications of selected NTEs, AlloDerm^™^ and Integra® had the highest publication number. The skin indications for AlloDerm^™^ are few compared with overall publication with other applications, reinforcing the hypothesis of proper market strategy to explore additional uses and invade other markets.

Two clusters attained maturity but differed in productivity, allowing speculation of the importance of the business model and clinical effectiveness as well as target product profile such as “off-the-shelfness.” Irrespective of this, both EMTs and CBTs have an ascending slope as their innovation continues. On the other hand, PBTs plateaued in 1992, suggesting that this cluster is on the path to senescence and, for further success, needs a technological jump, possibly innovation in immune tolerance. Senescence can be partially explained by a disruptive effect brought about by the rise of EMTs and CBTs—new technologies render older technologies obsolete.[4,48]

Scientific and business success are two interlinked but different concepts which depend on multiple factors (S1 Fig). The most successful SRTs are those who are not “end products,” for example Epicel^™^, and do not attempt to simultaneously solve all issues, rather address one particular aspect of skin replacement. Successful products allow space for further implementation and innovation. Success sometimes requires a change in paradigm, for example in the case of Integra® which instead of healing quicker, as initially projected, the healing lasted longer. Despite the benefits of dermis regeneration, it took two decades for the paradigm shift to occur before Integra® was used on a regular basis. Such versatility of use is a result of research and experimentation that together with proper business management may result in FDA approval for multiple uses that can potentially provide a high bar for competitors to achieve. An example of this is the PMA approval of Integra for burn, burn scar revision and diabetic foot ulcer.

For PBTs, success was finding better preservation methods of allografts and xenografts to increase shelf-life, decrease infection and demonstrate effectiveness. For CBTs, success was finding situations where rejection does not occur or does not play an essential role, as was the case with Apligraf® and Dermagraft®. Success for EMTs was achieved by finding the most efficient matrices and profitable ways of producing them, as well as enlarging the market.

In this analysis, we found that the level of innovation is a good performance indicator for technology. Indirectly this proves that SRTs are an innovation-based industry.

The database search strategy comes with limitations. Despite being extensive, Pubmed is not exhaustive and may lack literature published worldwide and older studies. As we excluded non-English language publications, as well as unpublished studies, this study is not free of publication bias. In addition, comparison of known historical milestones does not entirely correlate to the Pubmed search. For example, there is a discrepancy between the key time points of Reverdin performing the first skin allografts and the Pubmed search results. Also it is important to consider that “general meaning of words alter with time”[49] and lexicon of fields and terminology changes over time making it difficult to precisely isolate the publications on PubMed according to the subject of the search, neither it is required, affecting the model’s precision. The limitations of bibliometric analysis have been well defined, and include inefficiency of the text-based search method in selecting relevant publications as well as excluding irrelevant publications which contain the appropriate text strings [5]. The TIME model also suffes from inherent limitations. Specifically, the numerical methods it employs do not allow prediction of whether the technologies will exhibit a characteristic S-curve pattern of growth or when the exponential growth might slow.

There are also limitations in the assessment of the products worldwide, which was impossible and would be very difficult to compare based on no-similar regulatory and business development pathways. We attempted to be comprehensive however based on the resources we had we might have missed some products on the US market.

## Conclusion

The current study suggests that the main influence on success is clinical need with the potential to significantly improve a problem. The market-size must be large, and the product be first-in-class or at a stage before the cluster’s establishment point. In addition, the product should have room for further innovation or potential for diverse applications, must offer significant advantages over pre-existing products and be simple to produce in terms of materials, construction, and regulation. Products should be managed through an appropriate business model and market size and be released on the market when the economy and industry are ready. Compared to other fields of translational sciences which on average require 25 years from Te to production, our research emphasized that SRTs is much faster passed industry that has on average products appearing 4 years prior to Te for EMT and 11 years after Te for PBT, which makes this a very fast passed industry that could potentially be very attractive for investments. Overall, by analyzing TLCs, researchers, as well as industry, can appropriately maximize opportunity.

## Methods

### Data sources

Original publications and products of a technology were identified from 1900 to 2017. While performing the search, we noted a delay in reporting, finding new products and indexing in PubMed. Hence, and to decrease sampling error from missing new products and non-indexed publications, we set a study cut off for December 2015. Data regarding the publications were obtained from PubMed, while the database of products was compiled using the websites: accessgudid.nlm.nih.gov, www.fdazilla.com, www.FDA.gov, and www.510kdecisions.com, as well as insurance carriers’ websites BCBS, and reputable review articles on the subject. The publication and product search were conducted from January 1900 to end of 2017 and for the aforementioned reasons an earlier cutoff was set for December 2015. The specific search strategy and list of search terms applied when selecting eligible publications are available in S2 Fig and S1 File. The inclusion criteria for SRT products were any products available on the US market intended to be used for skin replacement or regeneration. All human cells, tissues, and cellular and tissue-based products that fall under sections 351 and 361 of the Public Health Service Act (PHS Act; 42 the United State Code) according to the Code of Federal Regulation, Title 21, Part 1271.20 and 1271.10, were included. The date of product appearance was considered when the tradmark was registered for the HCT/P 361 and for FDA regulated products the time of approval. All included products were approved for use in the US or are under different degrees of approval (S2 File).

### The bibliometric method, modulation of curves

The Technology Innovation Maturation Evaluation (TIME) model used in this analysis was first introduced by McNamee et al [5] and has previously been implemented in the field of gene therapy, Alzheimer’s disease, and cancer therapeutics [7,8]. Briefly, the technology analysis was performed by approximation of the exponential log-logistic regression to model the number of publications (N) as described in prior studies [7,8]:

$$N=L^{\left(\frac{1}{1+e^{-r(t-t_{0})}}\right)}$$

Calculation provides a logistic sigmoid function over log scales characterized by a symmetric growth phase that is exponential on average. The Initiation and Establishment points can then be calculated with:

$$Initiation/Establishment=t_{0}\pm \frac{a\;cosh(2)}{r}$$

Fig 1 with the technological milestones was prepared with Office Timeline (version 3.14, Bellevue, WA, USA) and subsequently modified by ACP. GraphPad Prism 5.0 and 7.0 and Microsoft Excel were used for analysis and figure preparation (S3 File).

## Supporting information

S1 Fig A. (top figure) “Sweet spot” of successful products. A successful product combines scientific advancement, ideal spatial-temporal context and proper marketing. B. (bottom figure) Technology performance machinery. Technological advantage implies increase in usage which drives increased in revenue and interest/attention which drived to even more increase in performance due to research.
(TIF)

S2 Fig SRT technological cluster Boolean term searches. (TIF)

S1 File Pubmed Boolean Searches–results of PubMed search. (XLSX)

S2 File New therapeutic entities–analyzed products. (XLSX)

S3 File Curve modulation–curve fitting. (XLSX)
