# Supplementary figures and images for: The life-cycles of skin replacement technologies
Source: PLoS One. 2020 Mar 4;15(3):e0229455. doi: 10.1371/journal.pone.0229455 (PMC7055911; doi:10.1371/journal.pone.0229455)

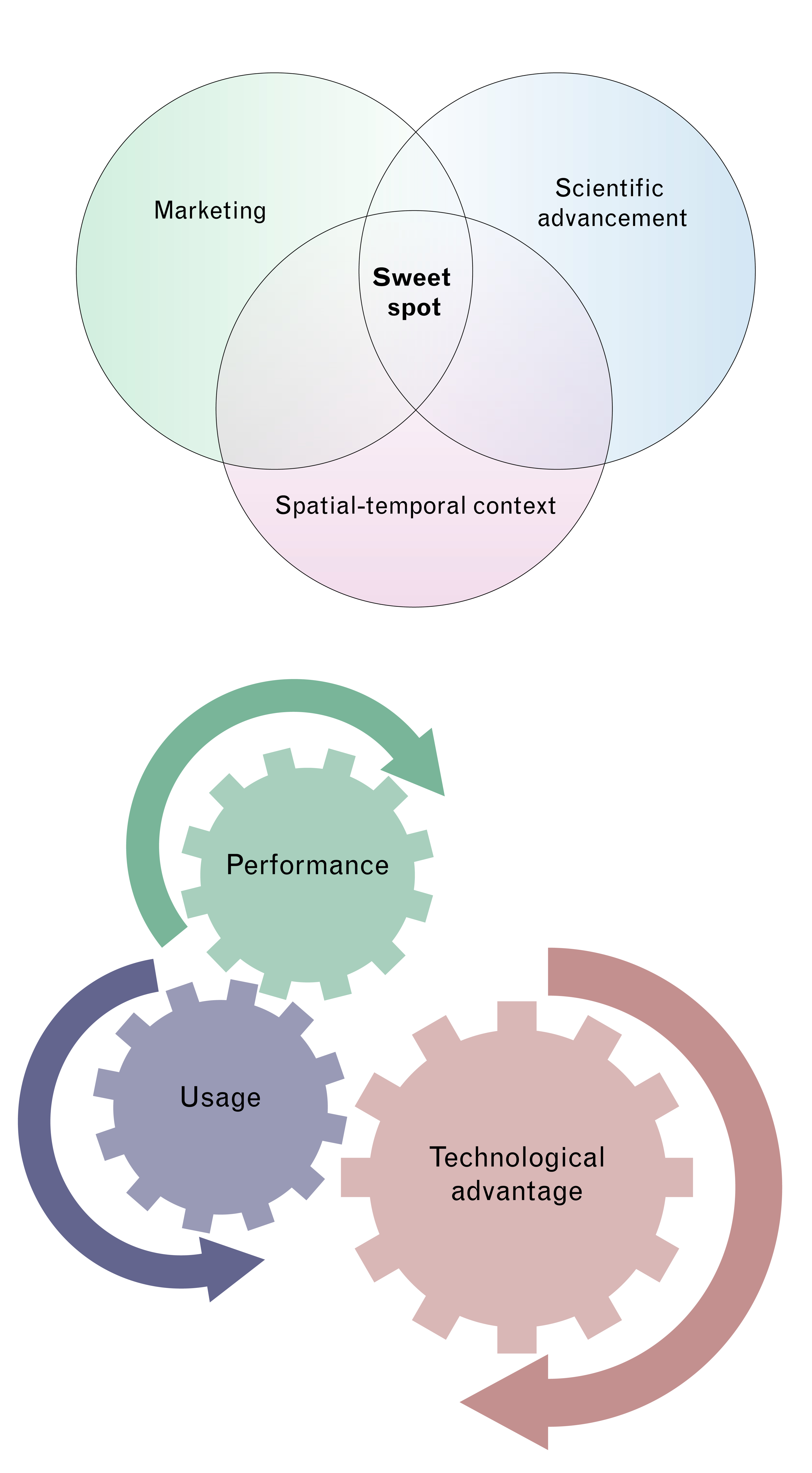

Supplement: S1 Fig — A. (top figure) “Sweet spot” of successful products. A successful product combines scientific advancement, ideal spatial-temporal context and proper marketing. B. (bottom figure) Technology performance machinery. Technological advantage implies increase in usage which drives increased in revenue and interest/attention which drived to even more increase in performance due to research. (TIF) [file pone.0229455.s001.tif]

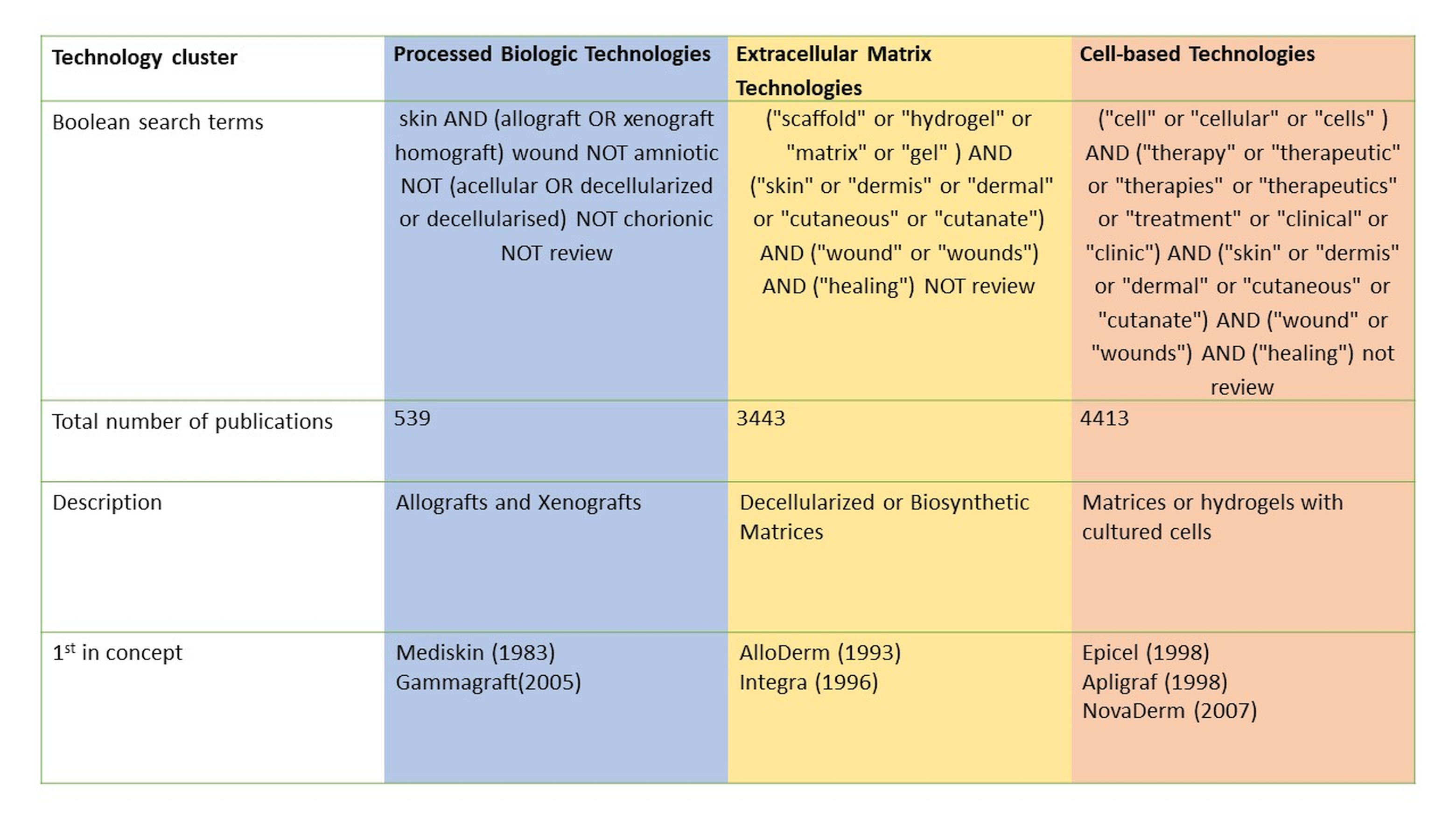

Supplement: S2 Fig — (TIF) [file pone.0229455.s002.tif]
